# Hansen’s Disease: A Practical Update on a Neglected Globally Significant Infection

**DOI:** 10.7759/cureus.57374

**Published:** 2024-04-01

**Authors:** Soukaina Benlamkadam, Amina Errahmany, Klevor Raymond, Mohamed Chraa, Najib Kissani

**Affiliations:** 1 Neurology, Mohammed VI University Medical Center, Marrakesh, Marrakesh, MAR

**Keywords:** world health organization, dermatology, neurology, endemic, leprosy

## Abstract

Leprosy is a great mimicker. It is caused by *Mycobacterium leprae* and *Mycobacterium lepromatosis*, together termed the *M. leprae* complex. Leprosy can result in systemic manifestations; however, the neurocutaneous syndrome is the most classic. There is a gap in recognizing the condition leading to misdiagnosis and delays in treatment. Leprosy remains an important cause of aesthetic and functional impairment. In this paper, we provide a practical review of leprosy touching on pathophysiology, clinical manifestation, classification, diagnostic approach and management of the condition in a way that can translate into clinical practice and help physicians better identify and manage potential cases of leprosy.

## Introduction and background

Leprosy is a chronic infectious disease caused by *Mycobacterium leprae *[1]. It is one of the oldest known infections in humans, becoming rare in modern times thanks to the efforts of the World Health Organization (WHO), but remaining mostly endemic in some regions of the world [2]. The WHO’s recent initiative 'Towards zero leprosy' is a forceful venture toward eliminating the condition definitely on a global scale by 2030 [3]. Though curable, leprosy remains an important cause of functional and aesthetic complications. Nevertheless, there is a gap in recognizing the condition which is rightly termed a 'great mimicker'.

The aim of this review is to provide a practical review of leprosy touching on pathophysiology, clinical manifestation, classification, diagnostic approach and management of the condition in a way that can translate into clinical practice and help physicians better identify and manage potential cases of leprosy.

## Review

Epidemiology

The number of new cases of leprosy recorded in 2022 was 174,087 worldwide representing a 19.3% decrease since 2013 [4]. The majority of cases in 2022 were found in India. Brazil and Indonesia also reported high incidences of leprosy [3]. It is important to note that data in 2022 are affected by the COVID-19 pandemic. As to whether the numbers truly reflect a fall in infection rates due to social distancing or rather a reduction in detection and reporting due to reduced access to healthcare remains to be clarified [5].

Causative agent, pathogenesis

Leprosy is caused by the alcohol and acid-resistant obligatory intracellular bacillus, *M. leprae* [1]. *M. leprae* was discovered by Gerhard-Henrik Armauer Hansen in 1873 in Norway, hence Hansen’s disease. In 2008, a group of scientists discovered a second species *Mycobacterium lepromatosis* responsible for a similar clinical phenotype as *M. leprae* [6]. Both species are termed together *M. leprae* complex.

The mode of transmission is through contact with infected skin and mucosa. Factors influencing transmission include host infectivity, proximity, duration and frequency of contact. *M. leprae *has a long incubation period ranging from two to five years with even longer durations reported [7]. Clinical symptoms are linked to direct damage of bacilli and to host immune reaction to the bacteria. *M. leprae *complex has a predilection for Schwann cells and the reticuloendothelial system; however, the clinical spectrum of leprosy is quite broad [8].

Clinical presentation

The clinical manifestations of Hansen’s disease can be attributed to the effect of chronic damage orchestrated by the bacilli and the immune response mounted by the host. This determines the disease phenotype and eventual classification. Leprosy is characterized by chronic clinical features and acute events corresponding to immune flare-ups called leprosy reactions [9].

Classically, leprosy causes a neurocutaneous syndrome consisting of hypopigmented or hyperpigmented macules or plaques, papules and nodules (lepromas) with reduced sensory testing over the skin lesions and motor weakness. While dermatologic lesions may be the sole manifestation of the infection, neurologic involvement may still be present at a subclinical level and may be revealed in electrophysiological studies. Hansen’s pure neuritis is a purely neurologic form of infection affecting the peripheral nervous system with no associated skin lesion [1].

**Figure 1:**
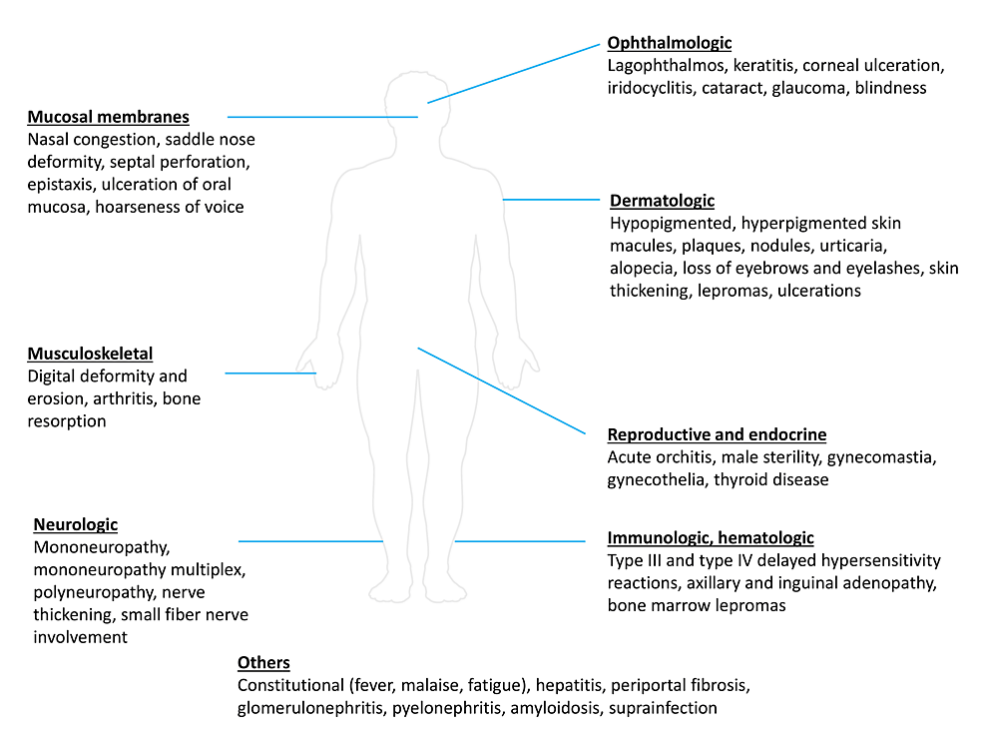
Clinical manifestations of leprosy Figure 1 is the original work of the authors. The figure shows the spectrum of clinical manifestations in leprosy [10-15].

Besides the skin and nerves, leprosy could affect any organ system either directly or through leprosy reactions or through complications of primary impairments. Symptoms could appear chronically or acutely. Figure 1 catalogues the spectrum of clinical manifestations of leprosy [10-15].

Diagnostic tests

The diagnosis is made based on the association of suggestive cutaneous findings, typically hypopigmentation, with peripheral nerve involvement, and confirmed on skin slit smears or skin or nerve biopsy. Nerve conduction studies are relevant to demonstrate peripheral nerve involvement electrophysiologically and the pattern of distribution of pathology [16]. It guides the choice of nerve biopsy site. This is also true for nerve ultrasound and nerve magnetic resonance imaging (MRI); however, their use in routine clinical practice is limited by issues of availability and the expertise required for their implementation. Nerve biopsy is considered the gold standard in the pure neuritic leprosy form [16]. It shows the underlying pathological process such as epithelioid granulomatous neuritis. Finding the acid-fast *M. leprae* on biopsy may be challenging. This is helped by performing quantitative polymerase chain reaction (qPCR) on biopsy specimens [1].

Classifications

Chronic Infection

Several historical classifications have attempted to categorize the broad phenotypes of leprosy. However, two are of relevance in the present: the WHO paucibacillary and multibacillary categories, and the Ridley-Jopling classification into five subtypes [17,18]. For all intents and purposes, the WHO classification has practical merits in deciding treatment regimens.

**Figure 2:**
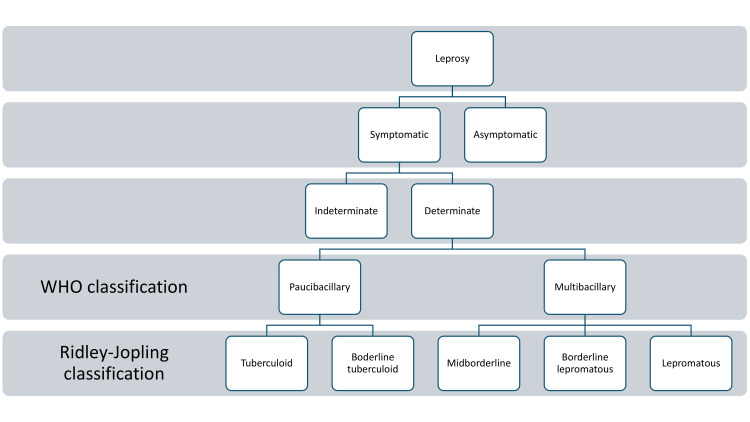
Tiered classification of leprosy Figure 2 is the original work of the authors. The figure shows a tiered approach to understanding the various classification systems of leprosy [17,18].

For the Ridley-Jopling classification, two polar phenotypes, the tuberculoid and lepromatous forms, are the anchors for the classification of other subtypes. These other subtypes are the borderline tuberculoid, the mid borderline, and the borderline lepromatous. These phenotypes differ in the number and type of cutaneous lesions, the general state of the skin, focal sensory impairment, and impaired hair growth. For example, the lepromatous form, contrary to the tuberculoid form, represents a form with a poor immune response resulting in numerous, disseminated, small skin lesions [7]. This form has a high bacillary index and thus would correspond to the WHO multibacillary category. The lepromin test is negative in this form of leprosy [18]. It is instructive to know that the paucibacillary form corresponds to a form with no more than five skin lesions. Typically, the bacillary index in this form is negative and corresponds to the tuberculoid and borderline tuberculoid forms according to the Ridley-Jopling classification system (see Figure 2) [17].

Acute Events: Leprosy Reactions

Leprosy reactions are acute or subacute events due to immunologic mechanisms which alter the progressive, chronic course of the infection. These reactions can occur before, during or after treatment of leprosy. They occur spontaneously but may be precipitated by emotional and physical stress, pregnancy, chemotherapy, concurrent infections or other comorbidities [7]. They are of two types. Type 1 leprosy reaction is a type IV cell-mediated delayed hypersensitivity reaction. It occurs in the Ridley-Jopling borderline forms of leprosy. It is characterized by inflammation of existing lesions and the emergence of new lesions. Neuritis is also observed, and patients present with sensory and motor symptoms. Type 1 leprosy reaction is termed 'reversal reaction' for the phenomenon of immunological ‘upgrading’ in which the organism mounts a stronger immune response, thus pushing the disease phenotype toward the tuberculoid pole. However, ‘downgrading’ could also occur transforming the phenotype toward the lepromatous pole [19].

Type 2 leprosy reaction corresponds to a type III immune complex-mediated delayed hypersensitivity reaction. This is also called erythema nodosum leprosum. It occurs in the lepromatous spectrum of leprosy. Patients present with erythema nodosum (painful subcutaneous nodules) with constitutional symptoms such as fever, fatigue, anorexia and malaise. They may also present multi-organ involvement including neurologic, testicular and joint involvement [20].

A peculiar form of type 2 leprosy reaction is the Lucio phenomenon which corresponds to a vasculitic necrotizing form of the condition [21].

Diagnosis and differential diagnoses

The WHO proposes the diagnosis of leprosy be made by finding at least one of the following cardinal signs:

(1) definite loss of sensation in a pale (hypopigmented) or reddish skin patch;

(2) thickened or enlarged peripheral nerve, with loss of sensation and/or weakness of the muscles supplied by that nerve;

(3) presence of acid-fast bacilli in a slit-skin smear [22].

However, several differential diagnoses should be considered when dealing with cardinal signs (1) and (2) (see Table 1) [23,24].

**Table 1:** Differential diagnoses of leprosy Differential diagnoses to consider based on various clinical phenotypes of leprosy [23,24].

Presentation	Differential diagnoses
Skin lesions	Pityriasis versicolor, pityriasis rosea, pityriasis alba, granuloma multiforme, lupus erythematosus, vitiligo, sarcoidosis, post-inflammatory hypopigmentation, mycosis fungoides
Peripheral neuropathy	Diabetic neuropathy, vasculitis, hereditary neuropathy with liability to pressure palsies (HNPP), infiltration with cancer or lymphoma, sarcoidosis
Thickened peripheral nerves	Chronic inflammatory demyelinating polyneuropathy (CIDP), Charcot Marie Tooth type 1A (CMT1A), hereditary neuropathy with liability to pressure palsies (HNPP), Refsum’s disease, amyloid neuropathy, neoplastic infiltration with tumours, neurofibromato

Management

Efficient management of leprosy should aim to prevent transmission, treat the infection, treat acute reactions, prevent drug toxicity, limit disability and fight stigma. This requires a holistic and multidisciplinary approach to the patient. The WHO recommends a supervised adherence to medication. The standard regimen is an association of rifampicin, clofazimine and dapsone. The duration of treatment is 12 months for the multibacillary form and six months for the paucibacillary form [22]. There has been a move from a two-drug regimen for paucibacillary forms to a unified three-drug regimen aimed at reducing relapse in patients with the paucibacillary form while averting undertreatment due to misclassification of multibacillary forms as paucibacillary [22].

High-dose steroids and eventually immunosuppressive therapies are required for the management of leprosy reactions. Management of peripheral neuropathy, in addition to the aforementioned treatment, may require antidepressants and antiepileptics to manage neuropathic pain. Surgical interventions may be required to repair disfigurement, decompress a nerve or transfer a tendon [25]. Physiotherapy, occupational therapy and psychotherapy go a long way to facilitate patients’ reinsertion into active social life. It is important for patients to be cautious during potentially traumatic activities and to ensure that they inspect their hands and feet daily to detect irritation. Patients must use correct footwear to prevent foot irritation. Eye dryness must also be avoided by face washing and 'think blinking', for example.

The treatment of contact cases is achieved by single-dose rifampicin [22]. Prophylaxis by vaccination is achieved by the bacille Calmette-Guérin (BCG) vaccine [26].

Outcomes and prognosis

Outcomes and prognosis are a function of the extent of tissue damage and functional impairment at the time of diagnosis and initiation of treatment. It is also a function of prompt diagnosis and appropriate treatment of acute events. Adherence is an important factor in ensuring good outcomes, hence, the recommendation of supervised medication by the WHO [22]. Drug resistance and adverse effects may limit drug use; however, the regimen of multidrug therapy and the possibility of replacing the standard drug regimen with ofloxacin, minocycline and clarithromycin allow for alternate options in case of issues with the standard regimen [22]. Recurrence or reinfection may occur thus requiring follow-up of patients even after treatment for early detection of cases [27]. Stigma can also limit reinsertion into active occupational and social life.

## Conclusions

Leprosy remains a global health issue. Prompt recognition of clinical manifestations, the classic neurocutaneous syndrome, is important for appropriate treatment in order to avoid complicated forms. Management should be holistic and seek, among other things, to combat stigma and insist on adherence to treatment. Future research should seek to make laboratory diagnosis more effective. Also, further work is required to create a specific vaccine for the condition. Finally, awareness-raising is crucial as the WHO has set its goal 'Towards zero leprosy' by 2030.
